# Dual-energy computed tomography material decomposition improves prediction accuracy of hematoma expansion in traumatic intracranial hemorrhage

**DOI:** 10.1371/journal.pone.0289110

**Published:** 2023-07-27

**Authors:** Jungbin Lee, Sung-Tae Park, Sun-Chul Hwang, Jung Youn Kim, A. Leum Lee, Kee-Hyun Chang

**Affiliations:** 1 Department of Radiology, Soonchunhyang University Bucheon Hospital, Bucheon, Korea; 2 Department of Radiology, Soonchunhyang University Seoul Hospital, Seoul, Korea; 3 Department of Neurosurgery, Soonchunhyang University Bucheon Hospital, Bucheon, Korea; 4 Department of Radiology, Cha University Bundang Medical Center, Seongnam, Korea; 5 Department of Radiology, Human Medical Imaging and Intervention Center, Seoul, Korea; University of Pisa, ITALY

## Abstract

**Objective:**

The angiographic spot sign (AS) on CT angiography (CTA) is known to be useful for predicting expansion in intracranial hemorrhage, but its use is limited due to its relatively low sensitivity. Recently, dual-energy computed tomography (DECT) has been shown to be superior in distinguishing between hemorrhage and iodine. This study aimed to evaluate the diagnostic performance of hematoma expansion (HE) using DECT AS in traumatic intracranial hemorrhage.

**Methods:**

We recruited participants with intracranial hemorrhage confirmed via CTA for suspected traumatic cerebrovascular injuries. We evaluated AS on both conventional-like and fusion images of DECT. AS is grouped into three categories: intralesional enhancement without change, delayed enhancement (DE), and growing contrast leakage (GL). HE was evaluated by measuring hematoma size on DECT and follow-up CT. Logistic regression analysis was used to evaluate whether AS on fusion images was a significant risk factor for HE. Diagnostic accuracy was calculated, and the results from conventional-like and fusion images were compared.

**Results:**

Thirty-nine hematomas in 24 patients were included in this study. Of these, 18 hematomas in 13 patients showed expansion on follow-up CT. Among the expanders, AS and GL on fusion images were noted in 13 and 5 hematomas, respectively. In non-expanders, 10 and 1 hematoma showed AS and GL, respectively. In the logistic regression model, GL on the fusion image was a significant independent risk factor for predicting HE. However, when AS was used on conventional-like images, no factors significantly predicted HE. In the receiver operating characteristic curve analysis, the area under the curve of AS on the fusion images was 0.71, with a sensitivity and specificity of 66.7% and 76.2%, respectively.

**Conclusions:**

GL on fusion images of DECT in traumatic intracranial hemorrhage is a significant independent radiologic risk factor for predicting HE. The AS of DECT fusion images has improved sensitivity compared to that of conventional-like images.

## Introduction

Traumatic brain injury (TBI) is a heterogeneous disease that ranges from intra-axial lesions, including diffuse axonal injury and hemorrhagic contusions, to extra-axial hemorrhages, such as subdural (SDH) and epidural hematomas (EDH). In these patients, non-contrast brain computed tomography (NCCT) is critical for triage because of its high speed, accessibility, and sensitivity to brain injuries [1]. When an intracranial hematoma is detected, the treatment plan is either to perform emergency surgery or conduct close observation. However, 16–65% of hematomas expand during follow-up, which may affect the treatment plan and prognosis [2]. Previous studies have revealed that the whirl sign on NCCT and the angiographic spot sign (AS) on CT angiography (CTA) are helpful for predicting hematoma expansion (HE) [3]. Unfortunately, despite the high specificity of these findings, their application is limited because of their relatively low sensitivity [4].

Dual-energy CT (DECT) is an imaging technique based on the principle that two X-rays of different energy levels have varying attenuation caused by a specific material, and it is used to distinguish the types of materials on CT [5,6]. In the field of neuroradiology, DECT has been helpful in differentiating contrast leakage from acute hemorrhage after endovascular treatment in acute ischemic stroke [7] and in identifying tumor bleeding from pure hemorrhage in intracerebral etiology [8]. Recent studies have reported that DECT is helpful in predicting the expansion of ICH and evaluating its prognosis [9,10]. However, these studies have limitations since they include heterogeneous etiologies, such as tumor bleeding and spontaneous ICH. Moreover, few studies have predicted hemorrhage expansion using DECT in extra-axial hemorrhages, such as SDH or EDH.

Therefore, the purpose of this study was to investigate the diagnostic performance of DE-CTA- AS for predicting HE in traumatic intracranial hemorrhage.

## Methods

### Study design

This retrospective study was approved by the Soonchunhyang University Hospital Institutional Review Board (IRB), and the need for informed consent was waived (IRB No. SCHBC 2021-08-027). Among the patients who visited our emergency room for trauma between December 2019 and April 2021, we recruited those who underwent brain CTA to screen for cerebrovascular injury based on the expanded Denver criteria [11]. They were included if intracranial hemorrhage was demonstrated. Patients were excluded if (a) the multiphase DECT scans were omitted, (b) the CT follow-up was impossible due to the patient’s condition (emergency surgery, transfer to another hospital, etc.), (c) the image quality was poor, or (d) only ineligible intracranial hemorrhages (subarachnoid hemorrhage/intraventricular hemorrhage, tentorial or subfalcine SDH, and scanty amount of hemorrhage; intra-axial <0.1 mL, extra-axial thickness <2 mm) were observed. Then, the following data of included patients were obtained from the medical records and PACS data server between October and December 2021: sex, age, CT follow up internal, past medical history, laboratory findings, mechanism of injury, physical and neurological findings, initial & follow up precontrast CT, and DECT angiography images.

### Data acquisition and post-processing

As part of the standard care for patients with suspected cerebrovascular injury, DECT angiography (DE-CTA) included precontrast, arterial phase, and venous phase (45-second delay) images. All DE-CTA images were obtained using a dual-source multidetector-row CT scanner (Somatom Definition Flash 128-slice dual-source scanner; Siemens Healthcare, Forchheim, Germany). DE-CTA (DE-CTA) images were acquired from the skull base to the vertex and underwent detailed image parameters of precontrast, arterial, and 45-second delayed phase images (100/140 kVp, 110/110 ref. mAs; slice thickness, 0.6 mm; dose-length product [DLP], 392 mGy cm). Patients were injected with 80–100 mL intravenous contrast media (Omnipaque, 350 mg/100 mL; GE Healthcare, Piscataway, NJ, USA). Based on the obtained dataset, conventional-like images similar to the usual 120 kVp single-energy acquisition were generated from precontrast, arterial, and venous phase images. The iodine map series and virtual noncontrast (VNC) images were reconstructed using a Brain Hemorrhage post-processing software (Syngo. CT Workplace 2012 B, Siemens Healthcare GmbH; Erlangen, Germany). Fusion images were generated by registration and overlay of the iodine map with conventional-like mixed images. Subsequently, a follow-up precontrast brain CT was performed within 72 h, depending on the patient’s condition, to evaluate HE.

### Imaging analysis

**Fig 1:**
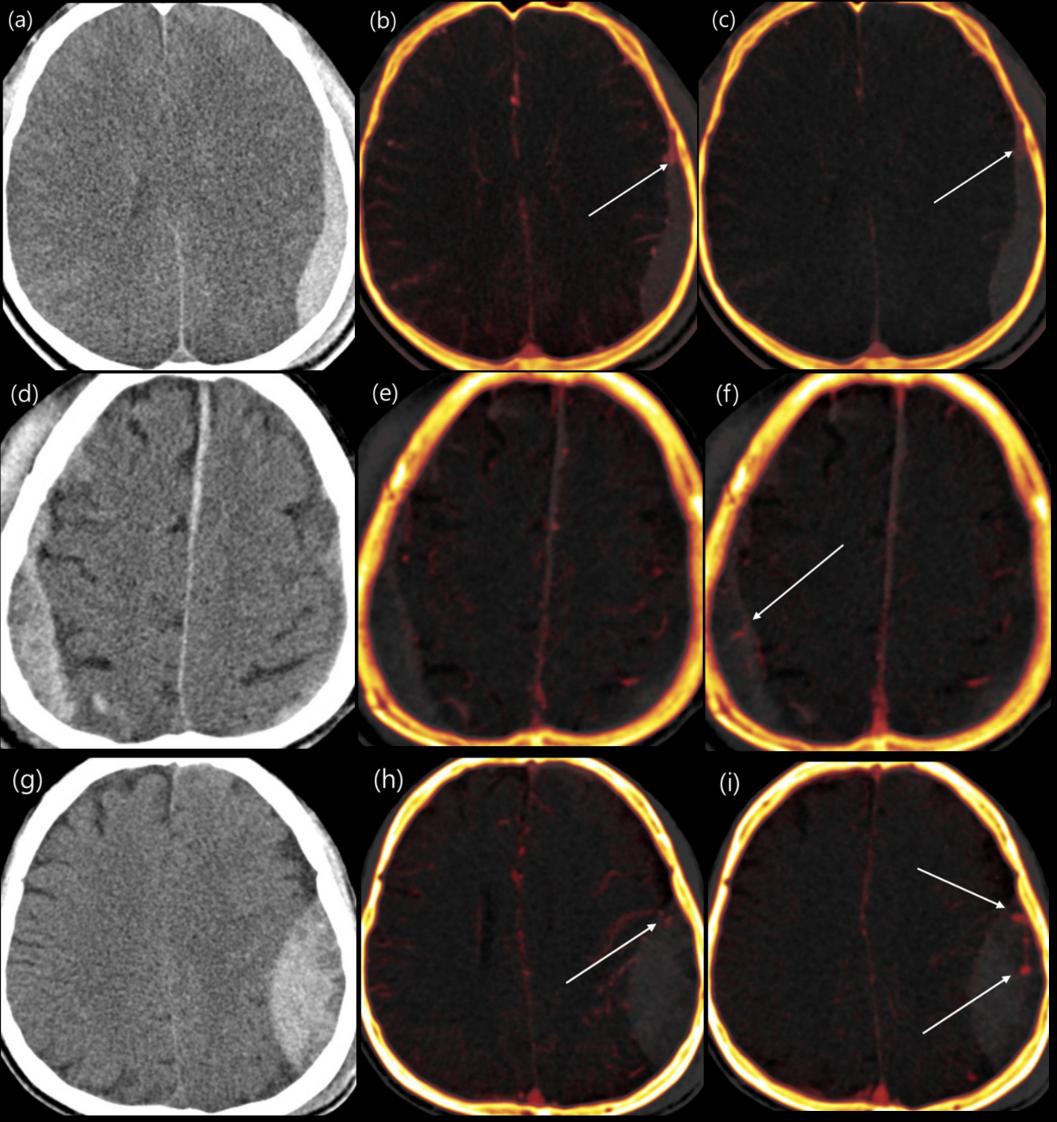
Classification of contrast leakage patterns. Dual-energy CT images (conventional-like images: a, d, g; arterial phase fusion images: b, e, h; venous phase fusion images: c, f, i) showing three contrast enhancement patterns: Intralesional enhancement without change (a–c), delayed enhancement (d–f), and growing contrast leakage (g–i).

Image analysis of all DE-CTA datasets was performed by a neuroradiologist with 10 years of experience (J.B.L.), without knowledge of follow-up CT findings and clinical status. The type of hematoma (traumatic ICH, SDH, or EDH) was classified on the precontrast CT included in CTA, and the amount of hematoma was measured using a semi-automatic method (Syngo.via). To ensure reproducibility of measurements, traumatic intracerebral hematoma was measured from >0.1 mL. Extra-axial hematoma was included in the measurement from >2 mm thickness, and falcine and tentorial SDH cases were excluded because the prognosis was reported to be significantly good [12]. Subarachnoid hemorrhage and intraventricular hemorrhage were not included in the analysis because of the possibility of redistribution in the cerebrospinal fluid space [13]. Using the iodine and VNC maps, we evaluated the presence of contrast leakage in the hematoma in the arterial and venous phases and compared the shape of the contrast leakage between the two phases. The contrast pattern was classified into three categories: intralesional enhancement without change (NC), delayed enhancement (DE), and growing contrast leakage (GL) (Fig 1). To compare fusion images and conventional-like multiphase images, the contrast leakage pattern in the hematoma was evaluated using the same method as fusion images on conventional-like arterial and venous phase images. Subsequently, the size of the hematoma observed on the follow-up NCCT was measured and compared to that on the initial CT. Then, the hematoma was classified as an expander or non-expander. Significant expansion of hematoma was defined as an increase of >6 mL in volume or an increase of >33% from the initial hematoma volume for both traumatic intracerebral and extra-axial hematomas [9]. In addition, to evaluate the interobserver agreement, two additional independent neuroradiologists (S.T.P. and J.Y.K., with 23 and 7 years of experience in neuroradiology, respectively) evaluated the image patterns on the fusion and conventional-like images in the same way; they were also blinded to the HE status and clinical outcome.

### Statistical analysis

In the per-patient analysis, to test the difference between the expander and non-expander groups, continuous variables were tested for normality using the Shapiro–Wilk test, and an independent *t*-test or Mann–Whitney U test was used according to the results. Fisher’s exact test was used to analyze categorical variables.

Interobserver agreement on the classification of contrast leakage patterns in the hematoma on fusion and conventional-like images was obtained using Fleiss-generalized kappa. The calculated kappa value was interpreted as follows: 0.01 to 0.2, slight agreement; 0.21 to 0.40, fair agreement; 0.41 to 0.60, moderate agreement; 0.61 to 0.80, substantial agreement; and above 0.80, almost perfect agreement [14]. In the per-lesion analysis, differences between the expander and non-expander groups in the size comparison between the initial and follow-up scans were analyzed using a *t*-test. Fisher’s exact test was used to determine the differences in the distribution of contrast enhancement patterns among the groups. Risk factor analysis for HE was performed using univariate and multivariate logistic regression. For multivariate logistic regression, the initial hematoma size and location (extra-axial or intra-axial) were corrected with a statistically significant difference set at *P*<0.05. Subsequently, the diagnostic performance and area under the curve (AUC) of the HE prediction model according to the contrast enhancement pattern were obtained, and the optimal cut-off value was calculated using the Youden index [15].

All statistical analyses were performed using MedCalc (version 14.8.1; MedCalc Software, Mariakerke, Belgium) and R statistical software (version 3.6.3; The R Foundation for Statistical Computing, Vienna, Austria). Differences were considered statistically significant at *P*<0.05.

## Results

### Study participants

Between December 2019 and April 2021, 41 patients underwent CTA in our emergency department to screen for cerebrovascular injury. Seventeen patients were excluded for the following reasons: no intracranial hemorrhage meeting the criteria (n = 11), inability to perform DECT analysis because of technical errors (n = 4), and absence of follow-up brain CT (n = 2). In total, 24 patients were included in the study, and their baseline characteristics are presented in Table 1. Among the patients with hematomas, 39 were included in the study. Hematoma location was intra-axial in 19 patients and extra-axial in 20. The average and follow-up hematoma sizes were 10.68±13.04 mL and 16.61±17.44 mL, respectively (Table 1).

**Table 1:** Baseline characteristics.

Demographic data				
Variable	All data (n = 24)	Expanders (n = 13)	Non-expanders (n = 11)	*P-*value
Age (years)	61.2±12.1	59.9±13.3	62.7±11.6	>0.05
Sex (male/female)	18/6	11/2	7/4	>0.05
CT F/U interval (hours)	8.4±7.7	5 (4.9–6.5)	6 (5–7)	>0.05
HTN (n)	11	8	3	>0.05
INR (second)	1.0±0.2	0.97 (0.95–1.08)	0.89 (0.86–1.01)	<0.05
PLT (count)	205.3±49.2	205 ±53.3	205.5±48.9	>0.05
Initial GCS score	14 (14–15)	14 (14–15)	14 (14–15)	>0.05
Antiplatelet/anticoagulation (n)	2	1	1	>0.05
Trauma mechanism				
Traffic accident	4	4		
Slip down	17	7	10	
Fall down	2	1	1	
Unknown	1	1		
Per-lesion analysis				
Variable	All data (n = 39)	Expanders (n = 18)	Non-expanders (n = 21)	
Initial size	10.68±13.04	11.72±12.03	9.46±14.39	>0.05
F/U size	16.61±17.44	21.45±21.12	12.46±12.64	<0.05
Enhancement without change	6	1	5	<0.05
Delayed enhancement	11	7	4	
Active contrast leakage	6	5	1	
Intra-axial/extra-axial	19/20	11/7	8/13	>0.05
Hemorrhagic contusion	18	10	8	
Hemorrhagic axonal injury	1	1	0	
EDH	6	1	5	
SDH	14	6	8	

Data are presented as the mean±standard deviation (SD), median (range), or n.

CT, computed tomography; F/U, follow-up; HTN, hypertension; INR, prothrombin time international normalized ratio; PLT, platelet; GCS, Glasgow Coma Scale; EDH, epidural hematoma; SDH, subdural hematoma.

### Contrast leakage and hematoma expansion

Of the 13 patients included in the study, 18 hematomas were observed to have significantly increased in size on follow-up CT. According to the demographic data, there were no significant differences between the expander and non-expander groups, except for the prothrombin time/international normalized ratio (PT/INR) (Table 1).

**Fig 2:**
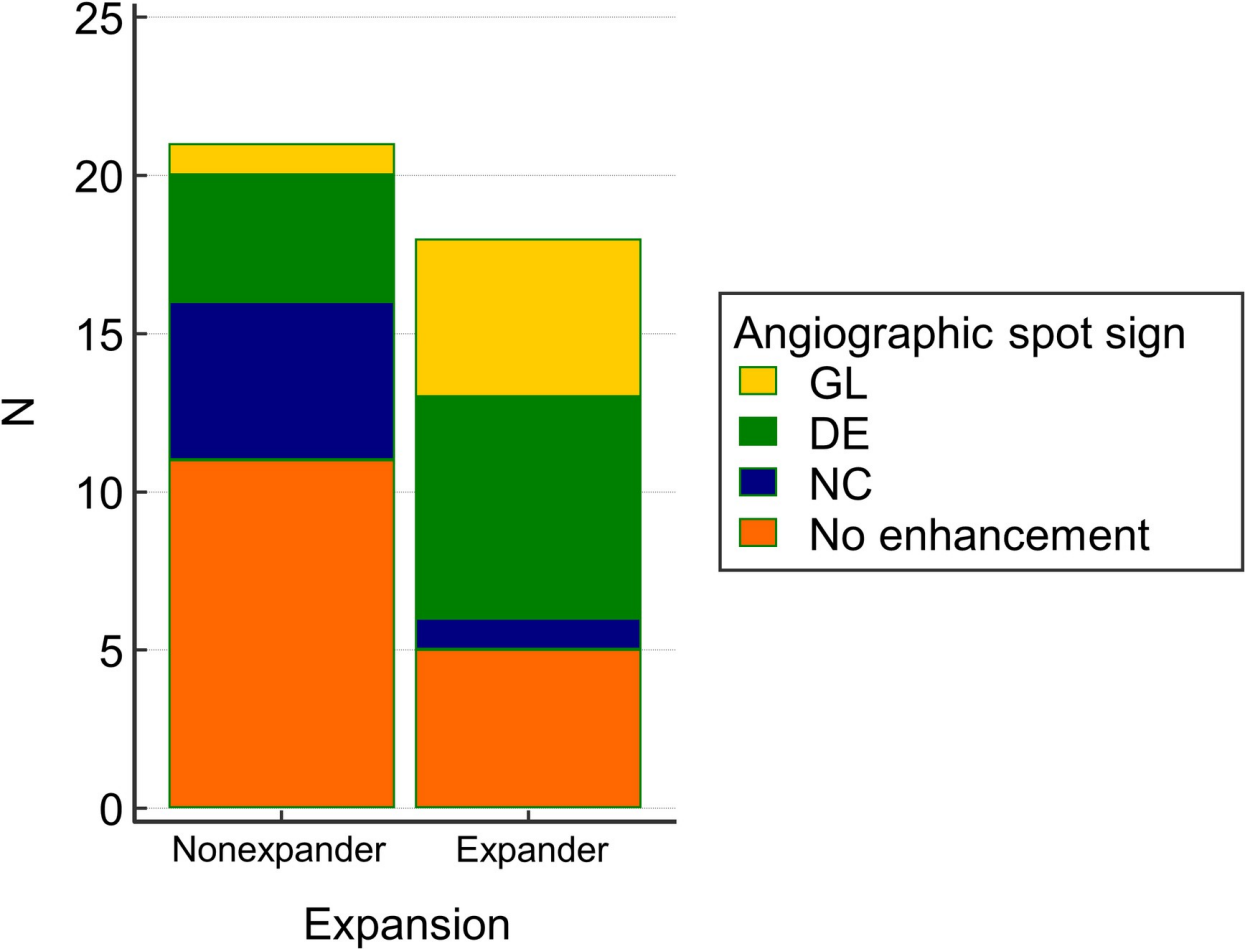
Distribution of contrast enhancement pattern by expander and non-expander groups. The ratio of growing contrast leakage (GL) and delayed enhancement (DE) in the expander group was higher than that in the non-expander group, and there was a statistically significant difference in the distribution between the two groups (Fisher’s exact test, *P*<0.05). NC, intralesional enhancement without change.

**Fig 3:**
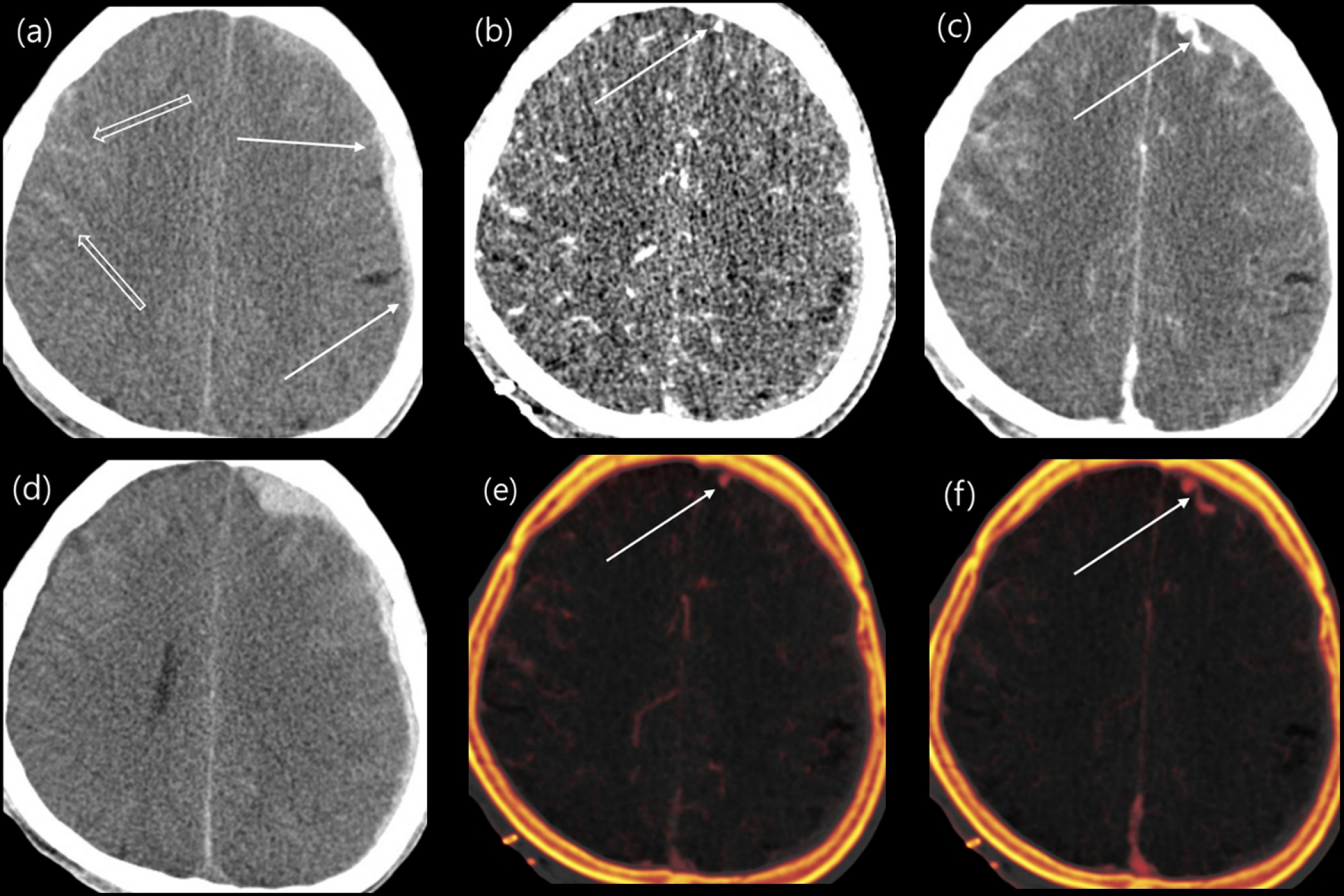
A representative case. A 50-year-old male patient visited our emergency department with a headache that occurred 30 min after a slip down before admission. On the initial precontrast brain CT (a), a thin acute subdural hematoma (SDH) was observed in the left frontal convexity (arrows), and an acute subarachnoid hemorrhage (open arrows) was observed along the right frontal cortical sulci. CT angiography was performed because of the possibility of cerebrovascular injury. Growing contrast leakage was observed in the arterial and venous phases of dual-energy CT (b, c, e, f). It was observed on both the fusion (e, f) and conventional-like images (b, c) but was clearly confirmed on the fusion images. On follow-up CT after 5 h (d), the amount of left frontal SDH was significantly increased.

Among all hematomas, intralesional contrast enhancement on fusion images of the DECT was seen in 23 of the 39 cases (59%), and the image pattern was as follows: NC, 6; DE, 11; and GL, 6. Intralesional contrast leakage on fusion images was observed in 13 of the 18 expanders (NC, 1; DE, 7; and GL, 5) and 10 of the 21 non-expanders (NC, 5; DL, 4; and GL, 1). A statistically significant difference was observed in the distribution (Fisher’s exact test, *P*<0.05; Fig 2). Logistic regression analysis was performed to identify the risk factors for predicting HE on follow-up CT; the location (intra-axial/extra-axial) and initial size of the hematoma were not significant; however, the GE of the fusion images was statistically significant (odds ratio [OR]: 11.0; 95% confidence interval [CI]: 1.00–120.44; *P*<0.05). Additionally, in the case of an adjusted model corrected for hematoma size and location, GL appeared to be an independent risk factor for predicting expansion (OR: 29.0; 95% CI: 1.67–502.9; *P*<0.05; Table 2). Fig 3 showed the images of HE on follow-up CT when the GL was observed on angiography.

**Table 2:** Univariable and multivariable logistic regression analysis for variables associated with hematoma expansion.

Variable	Univariate		Adjusted model (size and location)	
	OR (95% CI)	*P*	OR (95% CI)	*P*
Location (intra-axial/extra-axial)	0.39 (0.11–1.43)	>0.05	0.20 (0.03–1.47)	>0.05
Initial size	0.99 (0.94–1.04)	>0.05	1.00 (0.92–1.09)	>0.05
Contrast enhancement				
No enhancement	Baseline		Baseline	
NC	0.37 (0.03–3.91)	>0.05	0.93 (0.07–12.94)	>0.05
DE	5.13 (0.92–28.57)	>0.05	4.60 (0.76–27.90)	>0.05
GL	11 (1.00–120.44)	<0.05	29.02 (1.67–502.93)	<0.05

OR, odds ratio; CI, confidence interval; NC, intralesional enhancement without change; DE, delayed enhancement; GL, growing contrast leakage.

Meanwhile, when only conventional-like images were used, intralesional contrast enhancement was observed in 15 cases (NC, 1; DL, 12; and GL, 2). Contrast enhancement was observed in 10 of 18 patients (NC, 0; DL, 9; and GL, 1), and no statistically significant imaging findings were found to predict HE on conventional-like images.

The interobserver agreement between the image patterns of the intralesional contrast enhancement calculated for the fusion and conventional-like images was 0.80 (95% CI: 0.69–0.92) and 0.65 (95% CI: 0.51–0.78), respectively. This was perfect for the fusion image and substantial for the conventional-like image.

### DECT findings and diagnostic accuracy

**Fig 4:**
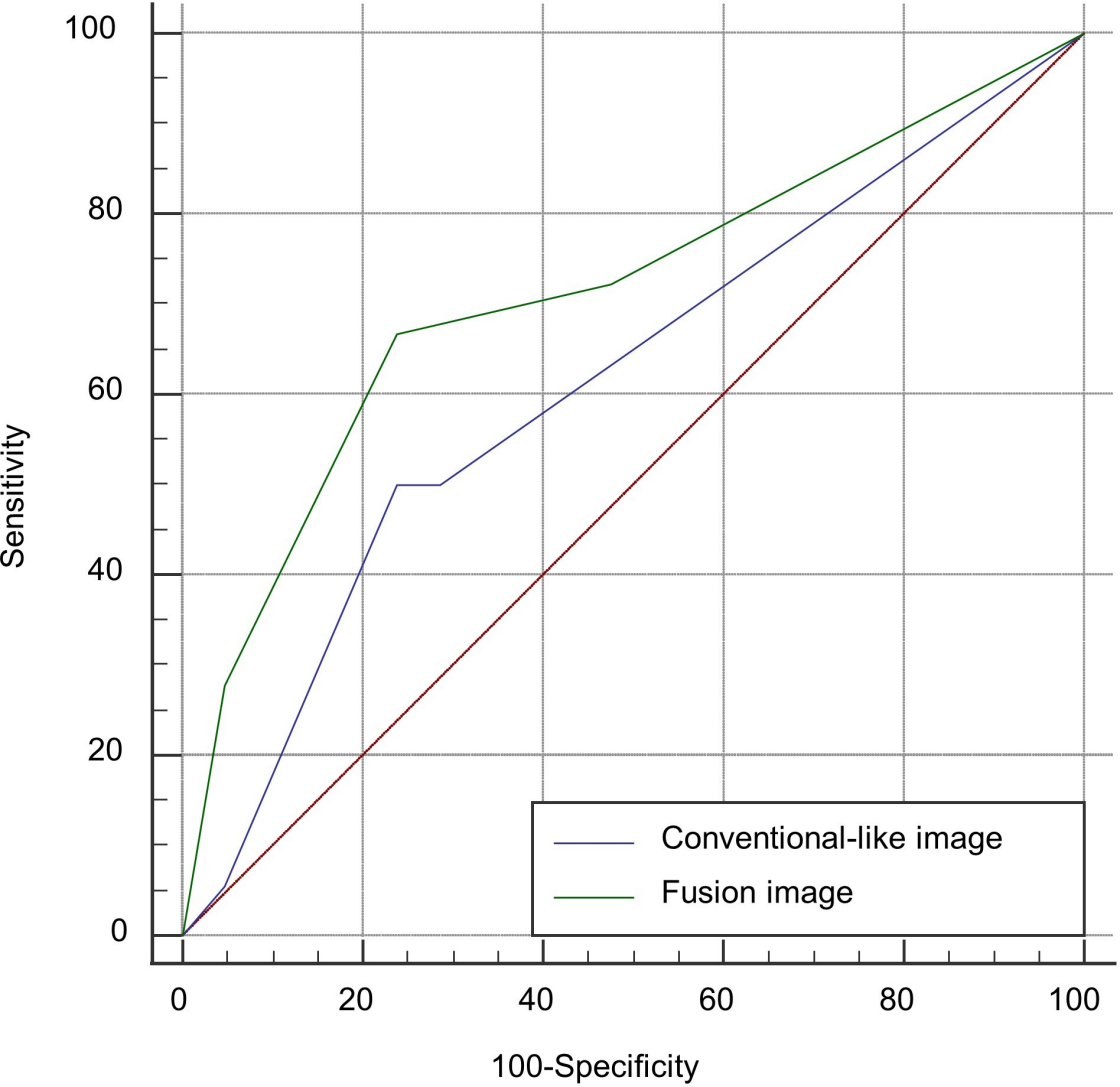
Receiver operating characteristic (ROC) analysis for prediction of hematoma expansion. Fusion images show a higher area under the curve than conventional-like images (0.71 and 0.61, respectively).

In the receiver operating characteristic (ROC) analysis for predicting HE using fusion images, the calculated Youden index was the highest (0.43) when DL and GE were included, where the sensitivity, specificity, and AUC were 66.7%, 76.2%, and 0.71 (95% CI: 0.55–0.85), respectively. When only GE was used for prediction, the sensitivity decreased to 27.8%, while the specificity was 95.2%. In contrast, in the case of the model using only conventional-like images, the calculated AUC was 0.61 (0.45–0.77), and the sensitivity and specificity were 50% and 76.2%, respectively (Table 3). The ROC curves of the prediction model using fusion and conventional-like images are presented in Fig 4.

**Table 3:** Receiver operating characteristic (ROC) analysis for prediction of hematoma expansion.

AUC comparison	AUC (95% CI)	
Fusion image	0.71 (0.55–0.85)	
Conventional-like image	0.61 (0.45–0.77)	
Cut-off points for fusion image	Sensitivity (%)	Specificity (%)
GL	27.8	95.2
GL+DE	66.7	76.2
GL+DE+NC	72.2	52.4
Youden index at GL+DL	0.43	

AUC, area under the curve; CI, confidence interval; GL, growing contrast leakage; DE, delayed enhancement; NC, intralesional enhancement without change.

## Discussion

In this study, among patients diagnosed with traumatic intracranial hematoma, GL in the hematoma on fusion images from dual-phase DECT was found to be an independent risk factor predicting HE, regardless of hematoma location and initial size. Furthermore, in the model for predicting HE using ROC analysis, the sensitivity, specificity, and AUC were 66.7, 76.2, and 0.71, respectively, showing higher sensitivity than when the conventional-like method was used. Additionally, in the interobserver agreement for the classification of contrast leakage findings, the fusion image showed a higher value than the conventional-like image.

Previous studies to predict HE using multiphase DECT showed improved predictive performance for intra-parenchymal hemorrhage resulting from various causes [9,10]. This study included extra-axial hemorrhages, including EDH and SDH, limiting the cause of the hematoma to traumatic hemorrhage. Similar to previous studies, DECT improved the prediction performance for HE independent of hematoma location. Furthermore, in a previous meta-analysis, the diagnostic performance of the AS of conventional CT was measured with a pooled sensitivity of 0.57 (0.49–0.64) [16]. The result of evaluating only the conventional image was 0.61, which was similar to the result of a previous study. In contrast, in the prediction model using DECT, the 95% CI was 0.71, a statistically significant improvement.

The low sensitivity of the AS is a major concern that prevents its use as an imaging biomarker in clinical trials [17]. In particular, it has been estimated that using a faster scanner with 128 slices or more is disadvantageous because the sensitivity of the spot sign decreases along with the scan time owing to the increased speed of the CT scanner [18]. In this study, particularly in the arterial phase, six AS were identified on fusion images out of the eighteen expanders, whereas only one true positive case was detected on conventional-like images. Compared to conventional-like images that detect contrast leakage inside the hemorrhage using only HU, fusion images are theoretically superior. Consequently, the DECT reconstruction software used in this study had a color-coded iodine map, which is advantageous for reader perception [19]. Therefore, it is presumed that the difference between DECT and conventional-like images increased in arterial-phase images with a relatively small lesion size. With these characteristics of DECT, the low sensitivity caused by the fast scan time of recent scanners can be overcome, and AS might be easily detected even in the emergency room, which is a relatively poor clinical setting for interpreting radiologic images.

In this study, among the imaging findings related to contrast leakage, only GL was a significant predictor of HE. In previous studies using multiphase conventional CTA, early phase images showed high specificity, whereas delayed images showed relatively high sensitivity by helping spot sign detection [18,20]. A recent study predicted that HE by DECT would also show this tendency, and that the sensitivity of both arterial phase and delayed phase images would improve on fusion images compared to that conventional-like images [9]. To date, the exact pathophysiological mechanism according to the timing of the radiologic spot sign has not been clearly elucidated [20]. In the case of the spot sign observed in the early phase, active leakage has been suggested [20]. In the delayed phase, it is thought to be extravasation sealed off by hemostasis [20] or related to a slower extravasation rate [2,21]. The results of this study are also in line with these assumptions. Furthermore, NC whose spot sign does not change in the arterial and delayed phases can be explained by a pseudoaneurysm. GL, which is observed in both the arterial and delayed phases and increases in size in the delayed phase, has the possibility of active contrast extravasation, whereas DL could be considered as having relatively slow extravasation, with or without sealing off by hemostasis [2].

Unlike many previous studies that included only ICH, this study also observed AS in extra-axial hemorrhages, such as SDH or EDH. Romero et al. [22] reported that the risk of HE and in-hospital mortality increased when contrast extravasation was observed in traumatic SDH. Another study that included SDH and EDH showed that contrast extravasation on CTA was associated with HE [2]. In this study, 20 extra-axial hemorrhages were included, and an increase in dots on dual-phase DECT was an independent risk factor for predicting HE. On conventional angiography, it is difficult to distinguish contrast leakage from curvilinear or dot-like cortical vessels overlapping with the high attenuation of the underlying hematoma. However, in the case of DECT, the morphology of the contrast enhancement can be visualized more clearly, making it easier to distinguish.

This study had several limitations. First, we used a virtual monochromatic image obtained from DECT as a conventional-like image instead of an additional single-source image. Obtaining single-energy CT for research purposes not only has ethical issues due to additional radiation exposure, but it is also known that virtual monochromatic images can obtain similar or better quality images without increasing the radiation dose compared to single-energy CT at 120 kVp [23]. In particular, one study showed that the contrast-noise ratio of iodine, which is the factor that has the greatest influence on the results of this study, is similar to or better than that of single-energy acquisition [24]. Therefore, the use of conventional-like images may have significantly affected the results. Second, this study was conducted with a relatively small number of patients, owing to the lack of established guidelines on the efficacy of multiphase DECT for traumatic intracranial hemorrhage. Furthermore, the use of contrast media for research purposes is ethically controversial. Therefore, this study retrospectively analyzed patients who underwent CTA for suspected traumatic vascular injury. The results of this study are expected to serve as a basis for future prospective studies. Third, the phase used for imaging consisted of an arterial and venous phase, which was not the phase optimized for AS. The purpose of this study was to determine the proper timing of AS. Therefore, researchers must optimize the timing to maximize the diagnostic yield of AS in subsequent studies. Finally, only a qualitative analysis was performed on AS. The iodine concentration in DECT can be calculated quantitatively; however, the CT source image must be preserved for this analysis [9]. This was a retrospective study, and because the source image of DECT was lost, only quantitative analysis could be conducted based on the reconstructed images at the time of patient management.

In conclusion, GL on fusion images of DECT in traumatic intracranial hemorrhage is a significant radiologic independent risk factor for predicting HE. As the AS of the DECT fusion image has improved sensitivity compared to that of the conventional-like image, it may have added value when applied to patients with traumatic intracranial hematomas, including SDH and EDH.

## Abbreviations

AS: Angiographic spot sign
AUC: Area under the curve
CI: 95% confidence interval
CNR: Contrast to noise ratio
CT: Computed Tomography
CTA: CT angiography
DE: Delayed enhancement
DECT: Dual-energy computed tomography
DE-CTA: Dual-energy computed tomography angiography
EDH: Epidural hematoma
GL: Growing contrast leakage
HE: Hematoma expansion
ICH: Intracerebral hemorrhage
NC: Intralesional enhancement without change
INR: International normalized ratio
NCCT: Non-contrast brain CT
PT/INR: Prothrombin time international normalized ratio
ROC curve: Receiver Operating Characteristic curve
SDH: Subdural hematoma
TBI: Traumatic brain injury
VNC image: Virtual noncontrast image
